# ‘They Just Let Us Rot to Death:’ Anti-Colonialism, Contestation, and Resistance to Reparations for Indian Residential School Abuse

**DOI:** 10.1177/09646639221138416

**Published:** 2022-11-14

**Authors:** Konstantin Petoukhov

**Affiliations:** 105732 University of Liverpool, UK

**Keywords:** Indian Residential Schools, contestation, resistance, settler colonialism, reparations, Independent Assessment Process, indigenous people, anti-colonialism, decolonisation

## Abstract

In the wake of increasing attention to reparations for settler colonialism in recent years, the politics of refusal and contestation of reparations has remained an underexplored area in socio-legal research. This article addresses this gap by foregrounding the perspectives of the colonised as a focal point to examine the strategies they mobilise to stage resistance to state-sponsored redress and to expose the harmful logics and legacies of ongoing settler colonialism. Strategies of resistance are discussed in the context of the Independent Assessment Process – a financial compensation process designed to provide redress to survivors of the physical and sexual violence they had suffered while attending Canada's Indian Residential Schools. This article explores how survivors disrupted the compensation process to advance an anti-colonial agenda, to politicise the violence, and to compel the settler state to recognise their lived experiences and realities of structural violence in the settler colonial present.

## Introduction

This article explores the strategies of resistance that victims of settler colonial violence mobilise to contest state-issued reparations. Contemporary debates on reparations fall predominantly into two distinct yet interrelated domains: one focuses on theorising the aims and objectives of reparations (Pradier et al., 2018) while the other draws analytical distinctions between various types of reparations (de Greiff, 2020). Aside from these debates, scholarly literature on reparations has paid little attention, with several notable exceptions (Brown, 2013; Jones and Brudholm, 2016; Moon, 2012), to how reparations recipients politicise, contest, and resist reparations. This gap is even more apparent in studies on reparations in nations with ongoing settler colonialism where reparations programmes often fail to recognise the linkages between historical and contemporary manifestations of settler colonial violence.

By approaching settler colonialism as both an historical *and* ongoing project, this article explores it as a structure rather than an event (Veracini, 2015; Wolfe, 2006) and examines the anti-colonial responses that victims put forward to expose the continuity of settler colonial violence. This analytical approach brings into question the limits of existing reparative responses, which are typically designed to address temporally discrete instances of *past* violence – a strategy that Indigenous scholars criticise as a ‘politics of distraction’ that constitute affirmative rather than transformative remedies and ‘reward colonial injustices’ (Corntassel and Holder, 2008: 472). The arguments that this article advances draw on settler colonial studies scholarship to situate power as a central feature of the settler colonial enterprise that dispossesses, marginalises, and subjugates the colonised. It is through this lens that the article critically examines individual material reparations – with a focus on financial compensation – as instruments of redress for settler colonial violence and their complicity in historicising and depoliticising injury, reinforcing the settler colonial status quo, and constructing wrongful acts as isolated events of the past rather than manifestations of an enduring and unjust social order. The article re-centres victim agency by exploring several themes in relation to resistance, counter-narratives, and transgressive politics that victims engage to contest the hegemony of state-sponsored reparations.

The article is divided into four sections. The first section theorises settler colonialism, examines individual material reparations for settler colonial violence, and explores anti-colonial responses to settler colonial measures of redress. The following section provides a brief history of Canada's Indian Residential School^1^ system and an overview of redress for the abuse that the children (survivors^2^) have suffered while attending the schools. The section on methodology outlines the research design and data sources used in the study on which this article is based. The analysis section is divided into three sub-sections that explore survivors’ truth-telling practices as strategies of resistance, collectivisation of individual compensation payments as process for exposing the structural violence of settler colonialism, and withdrawal and refusal to participate in the compensation process as acts of anti-colonial struggle for justice.

## Settler Colonialism, Anti-Colonialism, and Reparations

Settler colonialism is an invasive structure that seizes and occupies Indigenous territories where colonisers ‘come to stay’ and to ‘establish new political orders’ (Veracini, 2013: 313). At its core, settler colonialism is typically thought to encompass two interrelated logics: the *logic of exploitation and domination* and the *logic of elimination* (Wolfe, 2006; Veracini, 2011). The logic of exploitation and domination acts as a structuring element embedded within ‘interrelated discursive and nondiscursive facets of economic, gendered, racial, and state power,’ which reproduces and maintains unjust relations between the colonised and colonised through ‘the asymmetrical exchange of mediated forms of state recognition and accommodation’ (Coulthard, 2014: 6–7; 15). Under settler colonialism, the relationship between the coloniser and colonised is one of non-recognition or misrecognition and ‘reproduce[s] the very configurations of colonial power that Indigenous peoples’ demands for recognition have historically sought to transcend’ (Coulthard, 2007: 439). The colonised's demands for justice are typically met with concessions such as partial repatriation of traditional Indigenous lands, implementations of self-governance agreements, and accommodations of Indigenous interests that extend only so far as they do not challenge the settler colonial status quo (Nagy, 2022).

Through the logic of elimination, settler colonialism seeks to extinguish Indigenous peoples as distinct societies by destroying their political, legal, economic, and cultural systems (Veracini, 2019). Coupled with measures of exclusion, it ‘denies the identity of the colonised,’ subverts their resistance, and maintains access to Indigenous territories (Collins, 2011: 32). As Patrick Wolfe argues, ‘settler-colonisation is at base a winner-take-all project whose dominant feature is…*replacement*’ (Wolfe, 1999: 176, emphasis added). The logic of elimination advances settler colonial self-supersession that ‘erases the distinction between colony and metropole’ (Veracini, 2011: 3), upholds the principle of difference-blindness, and enables the settler colonial state to transform itself into a naturalised polity (Alfred and Corntassel, 2005).

The two settler colonial logics are interlocked in an apparent contradiction. On the one hand, the coloniser disavows the presence of the colonised; on the other, the coloniser calls the colonised into existence because ‘without the colonized, the colony would no longer have any meaning’ (Memmi, 1965: 66). From this perspective, the colonised occupy a precarious space defined simultaneously by destruction and continued marginalised existence, in which the colonising nation invokes indigeneity only to distinguish itself from the colonised and to ‘express its difference…from the mother country’ (Wolfe, 2006: 389). The settler colonial logics operate in synergy to facilitate the absorption of the colonised into settler colonial society and to grant the coloniser unquestionable power that precludes the contestation of the terms of unjust settler colonial relations (Coulthard, 2014). The process of settler colonisation is structured, in part, by ‘Othering,’ whereby the relationship between settlers and Indigenous populations is characterised not only by conquest, disavowal of Indigenous presence, and the establishment of a new political order, but also by the re-establishment and re-drawing of the contours of settler society. The distinction between the coloniser and colonised is constructed much in the same way as the binary opposition between the Occident and the Orient, in which the latter is constructed as inferior, uncivilised, and weak (Mamdani, 1998; Said, 1985). The binary opposition is maintained by means of the ‘otherisation’ of the colonised and legitimated through settler sovereignty, thus producing a ‘polity that seeks continually to fortify its legitimacy by marginalizing Indigenous claims’ (Balint et al., 2014: 203).

### Individual Reparations for Settler Colonial Violence

In recent years, Indigenous people in settler states have begun to launch claims seeking reparations for settler colonial violence. Reparations programmes are typically established to provide redress to victims of acute and extraordinary political violence, such as genocide and forced disappearances. They are often considered measures of recognition and acknowledgement that an injustice has, in fact, taken place and resulted in harm to victims (de Greiff, 2012). Financial compensation is one of several types of reparations that are normally deployed as modalities of redress for political violence (Balasco, 2017). It falls under the umbrella of individual material reparations that are issued directly to victims as cash payments or periodic pensions (Roht-Arriaza, 2014). A defining feature of financial compensation is that it is offered to victims who have *personally* experienced violence, rather than to collectivities of victims who have experienced violence by virtue of holding membership in a particular group^3^. Financial compensation programmes rely on calculation of damages inflicted on individual victims, which is an approach that requires precise identification of each victim and subsequent itemised accounting of compensable violence (Moffett, 2017). Despite the stated advantage of individual reparations to highlight the uniqueness of victims’ experiences, their application is problematic in contexts where identity-based violence targets victims as a collectivity (Walker, 2016). By disaggregating victims, individual reparations run the risk of individualising injustice, depoliticising it, and concealing the magnitude of atrocities.

While reparations programmes are most often implemented in nations undergoing transitions to liberal democratic regimes, recent years have seen a growing interest in the application of these transitional justice tools in stable settler colonial liberal democracies. Scholars caution that transitional justice in non-transitional societies may legitimate the settler colonial order by reinscribing a common national identity and ‘reinforcing the sovereign authority of the state over its Indigenous populations’ (Jung, 2011: 241). While transitional justice measures seek to induce a break from the violence of past political regimes and centre on reconciliation, peace, and justice in the present, the use of this model in ongoing settler colonialism has been criticised for its inability to recognise its enduring presence and for ‘purposely disentangling processes of reconciliation from questions of settler-coloniality’ (Coulthard, 2014: 108). By settling a dispute or resolving a complaint, the outcome of transitional justice measures implies that an injustice has ended and invites victims to move toward closure (Bentley, 2021). Consequently, transitional justice might impose an untimely resolution of the settler colonial condition in its attempt ‘to achieve the fantasy of the post-colonial condition and the settled future’ (Park, 2020: 268). This outcome might be at odds with victims’ own demands for justice when closure and finality are inappropriate in the wake of continuing violence that is expressed on the basis of ongoing plight of the colonised. Further, reparations in liberal democracies may serve as tools of a settler colonial state's efforts to pacify Indigenous struggles and to ‘channel Indigenous politics into a human rights framework as a way to undermine Indigenous demands for collective rights, on the one hand, and social and economic rights, on the other’ (Jung, 2011: 226). Thus, reparations may function as both products of and responses to settler colonial violence as they transition Indigenous victims to liberal subjects whose presence is circumscribed by ideologies of individual rights, individual autonomy, and private property – all of which are often thought to be harmful to the interests of Indigenous peoples (Logan, 2015; Slyomovics, 2014).

### Anti-Colonialism and Resistance to Settler Colonial Redress

An anti-colonial framework offers insight for exploring perspectives of the colonised on state-sponsored redress. Anti-colonial narratives centre on the epistemologies of the oppressed – their subjectivities, realities, and experiences – as tools of empowerment and resistance (Dei, 2017). While settler colonialism seeks to achieve self-supersession by erasing the traces of its presence and transforming itself into a naturalised order, anti-colonialism resists such closure by exposing the range of injustices that settler colonialism continues to inflict on Indigenous people, thereby blocking it from ‘covering its tracks’ (Veracini, 2013: 325). Anti-colonial strategies often target injustices associated with settler colonial dispossession, which are expressed in material and discursive forms (Coulthard, 2014). Anti-colonial movements strive to denaturalise the settler colonial enterprise and to combat settler colonial denial by exposing evidence of colonisation and highlighting the everyday realities of the colonised that uncover multiple loci of power and ‘work to establish dominant-subordinate connections’ (Simmons and Dei, 2012: 75).

Indigenous storytelling practices are often viewed as modalities of resistance, which act ‘as important processes for visioning, imagining, critiquing the social space around us, and ultimately challenging the colonial norms fraught in our daily lives’ (Simpson, 2011: 34). They represent political means of resistance and resurgence that are rooted in ‘Indigenous everydayness’ consisting of lived experiences of colonisation. Storytelling practices seek to indigenise previously colonised spaces and to ‘challenge the colonial status quo and attempted erasures of Indigenous peoples’ (Corntassel and Scow, 2017: 58). Ultimately, they represent the means of ‘evading the liberal politics of recognition's tendency to produce colonial subjects’ (Coulthard, 2014: 26). Stories may also create spaces of resistance that challenge and transform settler colonial power relations through ‘novel language, meanings, and visions of the future’ (Duncombe, 2002: 8). They act as performances that the colonised mobilise to deconstruct settler colonial narratives and construct ‘decolonial alternatives’ (Sium and Ritskes, 2013: VIII). Indigenous storytelling practices reflect what Canadian Indigenous scholar Glen Coulthard refers to as strategic desubjectification. Drawing on the work of anti-colonial political philosopher Frantz Fanon, the concept of desubjectification frames anti-colonial resistance as ‘practices of freedom’ that ‘critically revaluate, reconstruct and redeploy culture and tradition in ways that seek to prefigure…a radical alternative to the structural facets of colonial domination’ (Coulthard, 2007: 456). Desubjectification carries emancipatory potential aimed at triggering a shift away from looking ‘to that Other for recognition’ (hooks, 1990: 22) and toward identifying the ‘forms of structurally limited and constrained recognition conferred [to the colonised] by their colonial “masters”’ (Coulthard, 2007: 450). The process of desubjectification privileges anti-colonial epistemologies and ontologies and acts a medium for expression of the voices of the colonised whose resistance ‘asserts [their] presence in the face of erasure’ (Justice, 2008: 150).

To challenge the hegemony of settler colonial redress, the colonised might engage in ‘self-affirmative and self-transformative ethics’ that are rooted in the ‘establishment of relationships within and between peoples and the natural world built on principles of reciprocity and respectful coexistence’ (Coulthard, 2014: 48). In the Canadian context, I situate resistance strategies in relation to Courtney Jung's insights that Indigenous people may challenge the limited scope of state-sponsored redress, which applies primarily to acute and visible expressions of violence, to bring attention to ongoing structural violence, which is a product of unjust institutional arrangement and unequal power relations that typically manifest in the social marginalisation, political exclusion, and economic exploitation of the colonised (Jung, 2011). This approach draws broadly on John Torpey's concept of ‘anti-systemic reparations’ that target ‘economic inequalities rooted in a past system of domination’ to examine how the colonised may utilise reparations in a redistributive manner, seek to initiate social change, and trigger a shift toward egalitarianism by recognising the inequitable outcomes and life chances resulting from structural violence (Torpey, 2006: 65). These resistance practices constitute the colonised as active agents who assert their indigeneity and uncover the ‘link between conquest and dispossession, racialized power, and racialized privilege’ (Mamdani, 2001: 59).

Anti-colonial resistance to state-sponsored redress centres on the contestation of the authority of powerful actors who regulate and control the narratives of violence that ‘consign [them] to the dustbin of history’ and placate victims’ justice demands in the present (Spivak, quoted in Lawrence and Dua, 2005: 123). What is ultimately at stake in the acts of rejection or acceptance of reparations is the social significance of repair, which is interpellated with ‘historical, social and political’ meaning (Moon, 2012: 190). To bring attention to ongoing settler colonial violence, victims of settler colonialism might choose to ‘speak back’ by drawing on counter-discourses and counter-narratives that resist the officially sanctioned definitions of violence, harm, and repair, and by engaging in active reclamation of their ‘histories, traditions, and cultures against the subjectifying gaze and assimilative lure of colonial recognition’ (Coulthard, 2007: 453). Reparations can thus be mobilised as strategic spaces with a decolonising potential to advance outstanding justice demands associated with claims for recognition of the ‘social, political, economic and legal frameworks…that underlie settler colonialism’ (Balint et al., 2014: 214). These contestations of dominant narratives are made visible when the marginalised voices are ‘brought to the centre…[to expose] new truths about the past’ (Kempf, 2009: 27–28).

## Reparations for Indian Residential School Abuse

In the early 1800s, the Government of Canada established the Indian Residential School system, which was a network of schools designed to proselytise Indigenous children and to assimilate them into settler society by teaching them ‘to think, act, and believe as civilised Christians’ (Episkenew, 2009: 45). The schools, which persisted for nearly 150 years, were part of the settler colonial architecture aimed at eliminating Canada's Indigenous peoples as distinct societies (Neu and Therrien, 2003). They were funded by the Government of Canada and operated by the churches as boarding schools – total institutions – that housed students for as long as eleven months of the year.^4^ While attending the schools, students suffered various types of injustice, including malnutrition, inadequate medical care, and physical and sexual abuse perpetrated by school staff, including nuns, priests, and teachers. Several scholars have noted the genocidal tendencies of the schools (Powell, 2011; MacDonald, 2019) that they attributed to their ultimate objective to solve the ‘Indian problem’ by way of ‘absorbing Indian children into the body politic, until there would be no Indian question’ (McKegney, 2007: 85). The Indian Residential School system inflicted extensive and profound injuries on survivors and their communities, including intergenerational trauma, domestic violence, inability to parent, high rates of suicide, and widespread substance abuse (Bombay et al., 2013).

Throughout the 1990s and early 2000s, former Indian Residential School students began to launch individual and class action lawsuits against the Government of Canada and the churches alleging maltreatment that they had suffered while attending the schools. To stem the wave of approximately 18,000 lawsuits, the total claims of which threatened to bankrupt the defendants, the Government of Canada and the churches entered into negotiations with survivors^3^ (Truth and Reconciliation Commission of Canada, 2015). The negotiations resulted in a court-ordered settlement formally known as the *Indian Residential Schools Settlement Agreement*, which was ratified in 2006 (Indian and Northern Affairs Canada, 2006). The *Settlement Agreement* consisted of several components,^5^ including a financial compensation programme, officially known as the Independent Assessment Process (IAP), which launched in 2007 and concluded in 2020. The IAP – a quasi-judicial hearing process – was administered by the Indian Residential Schools Adjudication Secretariat and designed to adjudicate claims of sexual and serious physical abuse. The Secretariat screened IAP applications for eligibility and appointed Resolution Health Support Program (RHSP) workers to assist survivors though the emotionally difficult hearing process. The Secretariat encouraged survivors of abuse to seek legal representations from private law firms to prepare their applications. At the hearings, survivors provided oral testimony to the adjudicators who evaluated its credibility and reliability and determined the financial value of abuse and its consequences.

## Methodology and Research Design

In developing its arguments, the article draws on the research data collected as part of an exploratory case study (Berg, 2007; Yin, 2003) that involved 50 in-depth qualitative research interviews conducted in 2016^6^ in Indigenous communities, rural municipalities, and cities across Canada. Key informants in these interviews included IAP lawyers and adjudicators, RHSP workers, leaders of national Indigenous advocacy organisations, and survivors who applied for compensation under the IAP. The interview questions covered a wide range of topics, including the appropriateness of financial compensation for physical and sexual violence, the social significance and performative labour of compensation, and the potential of the IAP to act as a modality of settler colonial violence. Participants were selected using chain-referral and convenience sampling methods based on the initial samples of participants residing in the cities of Ottawa and Sault Ste. Marie, Ontario, Canada. The identities of all informants who participated in the interviews were anonymised, with several exceptions, and assigned aliases based on their respective genders.

The primary objective of the interviews with survivors was to learn about the IAP as a structured and structuring process. Drawing on the hermeneutic approach, which centres of the interpretation of meaning (Ricoeur, 1981), the interviews explored survivors’ first-hand experiences as participants in the application and hearing process, how they engaged in meaning-making in relation to their IAP compensation payments, and how they understood their role in relation to the other actors in the IAP, namely the RHSP workers, lawyers, and adjudicators. In the interviews with RHSP workers, the discourse analysis method was used to explore how their professional functions enacted, informed, and constructed discourses of victimisation and victimhood (Chouliaraki, 2008). These interviews provided insight into the role of RHSP workers as active agents who prepared survivors for their hearings, mobilised their professional capacity to provide health and cultural support, and shaped survivors’ experiences in the IAP. The interviews with leaders of Indigenous advocacy organisations explored the role of each organisation in advancing survivors’ legal demands and representing their interests in the negotiations of the *Settlement Agreement* and IAP. These interviews focused primarily on exploring the types of violence for which Indigenous-led organisations sought recognition and the extent to which the outcomes of the negotiations of the *Settlement Agreement* reflected survivors’ demands for justice. Research data from these interviews highlighted how the activities of Indigenous-led organisations enabled or inhibited dialogue surrounding compensable violence at the negotiations and the resistance they met from the legal representatives of the government and churches. Finally, the interviews with IAP lawyers and adjudicators served as the basis for generating insight into their functions as gatekeepers and regulators of survivors’ access to victim status. Responses from these interviews also contributed to exploring how the IAP empowered adjudicators and lawyers to construct discursive representations of survivors’ experiences and how their claims were adjudicated.

Although the article draws on the interview data from all four groups of participants, it centres primarily on the perspectives of survivors in its analysis and utilises data from the interviews with lawyers, adjudicators, RHSP workers, and leaders of Indigenous advocacy organisations to support and contextualise its arguments.

## Survivors’ Resistance Strategies in the IAP

The remainder of this article explores how Indian Residential School survivors exposed the continuity of settler colonial violence and expanded the restrictive definitions of harm to de-individualise financial compensation and to enable the recognition of collective and intergenerational harms in the IAP. As participants in the IAP, survivors animated transgressive politics to frame their Indian Residential School experiences as symptoms of ongoing settler colonial violence. By politicising the compensation process – the hearings and the financial awards they received – survivors contested settler colonial denial and the naturalisation of the rule of settler colonial law over Indigenous people (Cohen, 2001; Collins, 2011).

### Appropriation of the IAP Hearings as Spaces of Resistance

Survivors staged resistance by re-orienting themselves as political actors, reconstituting their indigeneity, and engaging in practices of self-affirmation and self-recognition at their IAP hearings. Within these strategies, practices of truth-telling positioned the Indian Residential School system within the spectrum of structural violence. Truth-telling practices can often be empowering and liberating experiences as they build counter-narratives of the marginalised and expose the violence and resulting harm that official measures of redress might fail to recognise (Lederach, 2005). Truth-telling practices challenge what Jacques Rancière refers to as the ‘police order,’ which encompasses an oppressive system of unjust ‘distribution and legitimization’ that builds false consensus (Rancière, 1999: 28). The police order demands that individuals and groups in society act and think according to their prescribed roles whose grammar is rooted in power structures that often go unquestioned (Davis, 2010). The police order is a violent structure insofar as it acts as a vehicle of exclusion of marginalises groups who fall outside the predetermined parameters and ‘who have no claim to contribute to the public discussion and debate, those who are, from the perspective of the police order, invisible’ (May, 2009: 112). In effect, their claims to justice do not count because they represent ‘those who have no part’ in the political arena (Rancière, 1999: 38). Theatrical dramatisation is one of the strategies of transgressive politics that resists the police order and draws on performative action aimed at (re)constituting the speaker as a political actor demanding recognition. Theatrical dramatisation transforms the space in which a previously invisible subject finds political existence by interpellating it with discourses that challenge the hegemony of the police order.

One powerful instance of truth-telling occurred at a time much earlier than the *Settlement Agreement*, when survivor Joseph Bonaparte launched an individual lawsuit against the Government of Canada for breach of fiduciary duty and damages including ‘loss of…parents’ guidance, care and companionship’ (*Bonaparte v. Canada*, 2003). The lawyer who represented Mr Bonaparte at a hearing recounted Mr Bonaparte's words in an interview with me, which are worth reproducing at length:

Lawyer: ‘Joe, what is it…what are you after? What do you want to have happen?’

Joseph Bonaparte: ‘I want to get into a courtroom and I want to get up in the witness box and I want to tell the Queen that she violated her treaty obligations with the Indian Nation.’

Lawyer: ‘The Queen's not going to be there.’

Joseph Bonaparte: ‘Yes she is. The Queen's judge is going to be there. And I want to tell my story to the Queen's judge and he’ll then tell the Queen’ (Ryan, IAP lawyer, 7 August 2016).

Mr Bonaparte constituted himself as an empowered speaker who, by means of producing a spectacle, publicised his legal battle before the media and the audience, and politicised his quest for justice against the Government of Canada. Mr Bonaparte's words depict an exchange in which a shift in the power relations between the sovereign and colonised has taken place. As a result of this shift, the coloniser was compelled to recognise the plight of the colonised, all the while bearing witness to his demands (Rancière, 1999: 28). Mr Bonaparte's act of truth-telling resembled an Indigenous principle of ‘witnessing,’ which acts as a process for remembering truth, affirming survivors’ experiences, and acknowledging the collective impact of Indian Residential School violence (Truth and Reconciliation Commission of Canada, 2015).

At the hearings, IAP adjudicators acted as representatives of the Crown and ‘the keepers of history’ who were appointed by the state to witness survivors’ testimony (Truth and Reconciliation Commission of Canada, 2015: 24). The very act of inviting survivors to hearings to tell their stories challenged a deep-seated denial of the past: ‘this is what I believe to be the truth because my ancestors told me that because their ancestors told them that’ (Matthew, survivor, 12 May 2016). Survivors often felt empowered by the fact that the IAP required the adjudicators to officially record their stories, which restored some of the dignity, recognition, and respect that has previously been withheld from them: ‘there's something about having people be obliged to listen to you’ (Richard, lawyer, 4 July 2016). Resistance took place at moments when survivor testimony transcended the prescribed boundaries of compensable violence: ‘Well you know what, this is important to me too, I want to talk about it because this is what happened and I want it down on paper’ (survivor's words recounted in an interview with Lisa, RHSP worker, 5 July 2016). By ‘speaking back’ at their hearings (Moon, 2012), survivors demanded recognition of what the IAP considered peripheral, contextual, and secondary factors in their claims for physical and sexual abuse: ‘I want the world to see that racism doesn’t work no matter what form you use’ (Del Riley^7^, survivor, 18 May 2016).

For some survivors, their IAP hearings were about restoring the equality that has deteriorated under settler colonialism: ‘I deserve an opportunity like everybody else. And I don’t care if you pay me a dollar or ten, you need to hear me’ (Jack, survivor, 15 June 2016). Survivors reported to have experienced a sense of relief when they had officially told their story for the first time in the presence of a powerful actor: ‘I finally told people in authority who were asked to judge our story, I finally told my truth. And it really didn’t matter if they believed it or not’ (Clyde, survivor and leader of an Indigenous advocacy organisation, 30 September 2016). Having their story officially acknowledged by an IAP adjudicator was often more important than receiving a compensation payment: ‘It wasn’t about money. I went there because of the acknowledgement of what happened to me. Not only was I abused, but I lost my language, I lost my identity, and I lost my culture’ (Jason, survivor, 30 July 2016). Survivors’ stories also identified the continuity of the genocidal tendencies of settler colonialism: ‘they just let us rot to death, I think that's what they had in their mind. As a matter of fact, it's still happening’ (Paul, survivor, 13 May 2016).

Survivors’ truth-telling practices challenged the permanence of their statuses as victims of settler colonialism that threatened ‘*colonization* to be the only story of Indigenous lives’ (Alfred and Corntassel, 2005: 601, emphasis in original). The process of shedding victim status incorporated elements of ‘survivance,’ which is a term that denotes an ‘active sense of presence, the continuance of native stories, not a mere reaction, or a survivable name’ (Vizenor, 1999: vii). Survivance carries emancipatory potential and empowerment that has enabled survivors to move beyond the violence they had suffered, to re-constitute themselves within their Indigenous cultural practices, and to reclaim their indigeneity and pre-colonial identities: ‘I’m a survivor…now I’m back to who I am. I’m a Cree guy, a Muskeg, you know, I speak my language, I do my ceremonies’ (David, survivor, 29 June 2016). As well, survivors’ truth-telling practices incorporated elements of desubjectification that denounced ‘an affirmative form of recognition from the settler-state and society’ (Coulthard, 2007: 456). Survivors chose to indigenise their hearings by actively integrating traditional Indigenous cultural practices and reclaiming indigeneity that the Indian Residential Schools sought to destroy: ‘when that cultural pride gets ignited within, oh I’ve seen people just shine’ (Michelle, RHSP worker, 11 May 2016). One survivor informed me he had requested to hold a circle hearing, which was inspired by Indigenous practices of sharing circles and storytelling that empower the speaker and facilitate the expression of truth based on lived realities and experiences (Sium and Ritskes, 2013). This type of hearing enabled him to locate his identity as a thriver and traditionalist: ‘I had a circle hearing, I made them sit around in a circle, and said, “This is who I am now, this is what the government at the time wanted to destroy, but I still have it, I still have my language, I still have my traditions, I still have my ceremonies”’ (David, 29 June 2016).

### Collectivisation of Financial Compensation Payments

Survivors staged resistance by contesting the official purpose of their IAP compensation payments, which was to provide redress for instances of physical and sexual abuse and consequential harm. They adopted counter-narratives that foregrounded the ‘social performance’ of their compensation payments in unexpected and remarkable ways that enabled the exposure of the collective dimensions of victimisation (Moon, 2012). In doing so, survivors built tangible linkages between their experiences as victims of institutional child abuse and the collective experiences of Indigenous families and communities as victims of colonialism in such a way that the distinction between individual and collective experiences became ‘immediately nonsensical at the moment of *reception* of reparation’ (Moon, 2012: 190, emphasis in original). By collectivising their compensation payments, survivors de-commodified their suffering and resisted the individualising gaze of the IAP that divorced their experiences of abuse from systemic economic maldistribution and racist ideologies (Million, 2020).

The individualising tendencies of the IAP compensation payments sparked comparative grievances between compensation recipients, on the one hand, and fellow survivors who did not qualify for an award under the IAP, on the other. This approach to compensation incorporated liberal ideologies that contradicted Indigenous worldviews that are often thought to be founded on principles of reciprocity, collective well-being, and shared resources:

[The IAP] drives wedges between the people that get the money and people that don’t have any money. It starts to become an ‘us and them,’ like a two tiered-community where they don’t share, then people start getting jealous, and if you look at the traditional values of our people, when somebody killed a moose, or killed a deer or an animal, then the whole community thrived because the whole community shared. The notion of not sharing within a community goes against the Seven Grandfather Teachings and is non-Indigenous (Pamela, RHSP worker, 15 September 2016).

Survivors understood their IAP awards in drastically different terms than how they were presented in the official discourses of abuse and harm in the *Settlement Agreement*. For many survivors, the primary motivation for participating in the IAP did not reflect the mandate of the compensation process to ‘resolve claims of abuse’ of so-called ‘direct victims’ (Indian Residential Schools Adjudication Secretariat, 2013: 3). Rather, survivors often regarded their IAP payments as anti-systemic reparations that recognised ‘group-based inequalities in the present’ (Torpey, 2006: 56). By re-distributing their compensation payments to their families and communities, survivors enabled the recognition of collective and intergenerational trauma of Indian Residential Schools and settler colonialism more broadly (Park, 2020). As one survivor whose IAP claim was rejected noted, he ‘would probably have spent it through the community. Communities need big help. That's why we’re here’ (Brian, survivor, 29 July 2016). Other survivors passed the payments onto their children as part of their ‘personal reconciliation projects’ that served as apologies for their inability to parent their children: ‘my actual amount was $152,000. So I divided it up, gave it to my kids. Cash. I said, I don’t know how to say ‘I’m sorry this happened,’ And they knew and understood’ (Jeffrey, survivor, 1 August 2016).

Collectivisation of individual compensation payments highlighted the urgent need for communal, not just individual, healing and challenged the liberal ideology of money as private property (Green, 1986). Money and wealth are often thought to be relational concepts in Indigenous communities: ‘[survivors] know the money has spirit, and the spirit of the money is intended to go around’ (Michelle, RHSP worker, 12 May 2016). In directing their compensation payments, survivors often consulted with members of their communities which resulted in collective decision-making involving knowledge keepers, traditional healers, and Elders: ‘what was I gonna do with it? Talked to my clan sister about it’ (Charles, survivor and Elder, 15 May 2016). Some survivors indicated that they had engaged in what Eva Mackey refers to as ‘relational healing’ (Mackey, 2013) that recognised the harm inflicted on the relational networks within Indigenous communities. To accomplish this task, survivors pooled their compensation payments to establish community healing events for all survivors, including intergenerational survivors^8^, regardless of their participation in the IAP. Survivors directed their IAP awards to address the socio-economic disadvantages that their children had faced growing up: ‘90% of [survivors] didn’t keep a penny of that, they gave it to all to their children. And these people, they lived in poverty their whole entire lives, and they’ve never been able to give their children anything’ (Linda, RHSP worker, 13 June 2016).

### Resisting the IAP by Withdrawing and Refusing to Participate

Survivors resisted the harmful ideologies of the compensation process by refusing to participate in the IAP or withdrawing from the *Settlement Agreement* entirely (Popic, 2008). Refusal to participate in a reparations process represents a political gesture that contests the power of the state to draw boundaries around compensable violence and to expose reparations as tools of social control (Moon, 2013). Acts of refusal resist untimely closure that reparations seek to ensure by ‘consigning horror to historical memory’ and pacifying debates about justice in the present (Moon, 2012: 197). By this logic, objections to the IAP appeared to resist its mandate to settle claims of historical injustice, thus seeking to enable the recognition of Indian Residential School abuse on the continuum of ongoing settler colonial violence. As the IAP positioned the Indian Residential School system as a *synecdoche* of settler colonial violence, which is a process where a part comes to represent the whole (Henderson, 2013), it perpetuated the narrative that once ‘the historical ‘wrong’ has been ‘righted’…further transformation is not needed’ (Simpson, 2011: 22). To accept reparations would mean to accept a particular political narrative and version of the past that would violate survivors’ interpretations of the events.

In total, an estimated 340 former students withdrew from the *Settlement Agreement*, one of whom was Delbert (Del) Riley^9^. In response to the IAP's lack of recognition of violence beyond the physical and sexual abuse, Mr Riley elected to develop his own compensation model and pursued legal action against the Government of Canada and churches by means of an individual lawsuit. For Mr Riley, the IAP represented a partisan and unfair process that was unilaterally established by the Government of Canada, which stacked the odds against survivors and positioned the government both as the perpetrator of violence and dispenser of justice: ‘everything was internal: the government was the judge, jury, and executioner’ (Del Riley, survivor, 17 May 2016). Indeed, as an RHSP worker pointed out, ‘individuals who were basically abused quite badly by one federal system, an extension of the federal system that the lawyer works for’ (Pamela, RHSP worker, 15 September 2016). Frustrated with the IAP, other survivors chose to launch individual lawsuits against their perpetrators: ‘I’m going to take you to court and I’ll prove that you did that to me’ (Michael, survivor, 17 June 2016).

Mr Riley's motivations to reject the IAP included its non-recognition of the intergenerational, long-term impact impacts of Indian Residential School abuse on his children (and on Indigenous families and communities more generally), breach of trust, breach of fiduciary duty, and cultural genocide: ‘here's the problem with the IAP: they had about 4–5 items you could use. I figure I’ve got 70 or 80 agenda items to discuss and to put a price on’ (Del Riley, survivor, 17 May 2016). Ironically, the items in Mr Riley's statement of claim resemble those found in several older class action lawsuits, including *Cloud* and *Baxter* (*Baxter v. Canada, 2006*; *Cloud v. Canada, 2004*) that the *Settlement Agreement* sought to address. Indeed, at the negotiations of the *Settlement Agreement* and the IAP in particular, the Government of Canada's opposition to recognise the mandate of the Indian Residential Schools to commit genocide through assimilation was indicative of denial and depoliticisation of violence: ‘the word “genocide” was not welcome on any governmental level, they refused to accept that word’ (James, survivor and leader of an Indigenous advocacy group, 7 June 2016). Accordingly, Mr Riley's lawsuit appears to have challenged the ‘statist forms of recognition’ and performed a generative function that brought attention to the previously hidden histories and reframed the previously non-justiciable violence (Simpson, 2007: 78). His refusal could be characterised as a critique of the limited scope of reparations, which is a politically significant act that ‘*refuses* the authority of the state’ (Simpson, 2007: 74, emphasis in original).

Other survivors had chosen to engage in resistance strategies that did not go so far as opting out of the *Settlement Agreement*, but rather encompassed non-participation in the IAP either by forfeiting their right to file a claim or by withdrawing their claims in progress. Survivors’ reasons for refusal to participate in the IAP were diverse. In their previous encounters with the Canadian criminal justice system, many survivors had developed an aversion to the ‘political structures and judicial system’ that had often revictimised and discriminated against them (Jessica, RHSP worker, 27 June 2016). Evidently, survivors’ reluctance to participate in the IAP stemmed from their deep-seated distrust of a process established by ‘White people. And survivors listened to it and it just sounded like another White person process and they’d be hurt again’ (Sarah, IAP lawyer, 8 June 2016). For them, the IAP represented just another instance in the history of broken promises that they viewed as ‘one big disappointment in the gap between what's promised to them and what they actually get. And that's something that they lived with their entire lives: “No, I just don’t believe that this is going to make my life any better”’ (Sarah, lawyer, 8 June 2016). In addition, survivors decided not to launch compensation claims because they felt that the IAP had not been designed to account for non-compensable harms such as depression and loneliness that the Indian Residential Schools were responsible for: ‘they didn’t want to know my story and they just refused my claim’ (Susan, survivor, 29 June 2016). For others, the IAP represented an unfair compromise that sought to silence survivors by requiring them to sign a legal document at the conclusion of their hearings that would absolve the Government of Canada and churches of any additional responsibility toward survivors: ‘you sign off that you’re not gonna sue them ever again. And that's all they wanted’ (Matthew, survivor, 11 May 2016).

## Concluding Remarks

As a modality of financial compensation, the IAP represented an imperfect system that occupied a paradoxical space. On the one hand, the IAP could never adequately and fully compensate survivors of Indian Residential School abuse because it was unable to account for the totality of their experiences; yet, on the other hand, it is important to recognise that leaving survivors uncompensated was not an option. Survivors transformed several components of the IAP into spaces of resistance by framing their experiences of abuse as modalities of settler colonial violence. Their resistance strategies challenged the individualising tendencies of the IAP compensation payments and expanded their meaning to bring attention to instances of structural violence that financial compensation is normally incapable of recognising. Survivor testimony played a central role in the staging of resistance by positioning truth-telling practices, politicising the objectives of the Indian Residential School system, and re-establishing indigeneity by re-valuing it as an essential cultural attribute. Refusal to participate in and withdrawing from the IAP constituted gestures of disobedience and non-compliance with the harmful settler colonial logics that threatened to revictimise survivors, failed to recognise the broader impacts of the Indian Residential Schools, and perpetuated the denial of its persistent drive for the ‘elimination of the Native’ (Wolfe, 2006). In many ways, resistance strategies sought to re-balance the power relations between the settler colonial state and Indigenous people, to reclaim precolonial Indigenous identities, and to seek redress for past injustices while recognising their ongoing legacies in the present.

Survivors’ accounts of resistance and contestation invite us to reconsider the implications of deploying individual material reparations as responses to settler colonial violence. Whereas reparative responses focus primarily on past injustice characterised by acute, extraordinary, and visible violence, settler colonial violence (much of which is structural) resists historicisation as it transcends the boundaries of the past and continues into the present. Accordingly, temporally discreet manifestations of settler colonial violence must be understood in the context of its logics of domination, oppression, exploitation, and elimination of Indigenous peoples as distinct societies. Anti-colonial resistance exposes settler colonialism as a naturalised order and links personal experiences of the colonised with structural injustices in the process of unmasking and unmaking of settler colonialism (Nagy, 2012). By mobilising strategies of resistance and contestation, victims of settler colonial violence constitute themselves as active agents of change who declare their political subjectivity and highlight the urgent need for decolonisation.

## Notes

1. The term ‘Indian’ has long been replaced with the widely accepted term ‘Indigenous’ to identify the First Peoples of Canada. The *Indian Act* and the *Canadian Constitution* continue to refer to the First Peoples as Indians within their respective legal contexts.

2. Former Indian Residential School students are commonly known as “survivors.”

3. In the reparations literature, group membership is often defined based on political, ethnic/racial, or religious affiliation, as evident in case studies on Argentina's Dirty War, South African Apartheid, and in the Guatemalan Civil War. See Arthur P (2011) *Identities in transition: Challenges for transitional justice in divided societies.* New York: International Center for Transitional Justice.

4. For a comprehensive overview of the Indian Residential School system, see Miller JR (1996) *Shingwauk's vision: A history of native residential schools.* Toronto: University of Toronto Press.

5. In addition to the IAP, the *Settlement Agreement* also contained the Common Experience Payment (CEP) programme that was designed to compensate eligible former students for their overall negative experience in the Indian Residential School system. As well, it established the Truth and Reconciliation Commission of Canada, which completed its work in 2015 and was mandated to uncover the truth about the schools and to ‘pave the way to reconciliation’ between Indigenous and non-Indigenous Canadians. The *Settlement Agreement* also allocated funds for the Aboriginal Health Foundation that sought to establish healing programmes that would address the legacy of Indian Residential School abuse. Finally, the *Settlement Agreement* established a commemoration fund to ‘honour, educate, remember, memorialise and/or pay tribute to residential school former students, their families and their communities and acknowledge their experiences and the broad and systemic impacts of the residential school system’ (Indian Residential Schools Settlement Agreement, Schedule ‘J’).

6. In total, I interviewed fifteen Indian Residential School survivors, fifteen RHSP workers, four leaders of Indigenous advocacy organisations, and sixteen IAP lawyers and adjudicators. The interviews were conducted as part of doctoral fieldwork in 2016 and the interview data continue to be disseminated in the form of scholarly articles and book chapters.

7. In this instance, I use the survivor's real name at his request.

8. Intergenerational impacts of abuse include ‘the consequences of numerous and sustained attacks against a group [that] may accumulate over generations and interact with proximal stressors to undermine collective well-being’ (Bombay et al., 2014: 320).

9. Delbert Riley is former Chief of the Assembly of First Nations, which is the governing body of Canada's First Nations.
